# Parkinson’s disease with restless legs syndrome—an in vivo corneal confocal microscopy study

**DOI:** 10.1038/s41531-020-00148-5

**Published:** 2021-01-05

**Authors:** Mattias Andréasson, Neil Lagali, Reza A. Badian, Tor Paaske Utheim, Fabio Scarpa, Alessia Colonna, Stephan Allgeier, Andreas Bartschat, Bernd Köhler, Ralf Mikut, Klaus-Martin Reichert, Göran Solders, Kristin Samuelsson, Henrik Zetterberg, Kaj Blennow, Per Svenningsson

**Affiliations:** 1 Center for Neurology, Academic Specialist Center, Stockholm, Sweden; 2 Department of Neurology, Karolinska University Hospital, Stockholm, Sweden; 3 Department of Clinical Neuroscience, Karolinska Institutet, Stockholm, Sweden; 4 Department of Biomedical and Clinical Sciences, Linköping University, Linköping, Sweden; 5 Unit of Regenerative Medicine, Department of Medical Biochemistry, Oslo University Hospital, Oslo, Norway; 6 Department of Ophthalmology, Oslo University Hospital, Oslo, Norway; 7 Department of Information Engineering, University of Padova, Padova, Italy; 8 Institute for Automation and Applied Informatics, Karlsruhe Institute of Technology (KIT), Karlsruhe, Germany; 9 Department of Clinical Neurophysiology, Karolinska University Hospital, Stockholm, Sweden; 10 Institute of Neuroscience and Physiology, Department of Psychiatry and Neurochemistry, Sahlgrenska Academy, University of Gothenburg, Mölndal, Sweden; 11 Clinical Neurochemistry Laboratory, Sahlgrenska University Hospital, Mölndal, Sweden; 12 UCL Institute of Neurology, Department of Neurodegenerative Disease, Queen Square, London, UK; 13 UK Dementia Research Institute, London, UK

**Keywords:** Parkinson's disease, Parkinson's disease, Parkinson's disease, Basal ganglia

## Abstract

Small fiber neuropathy (SFN) has been suggested as a trigger of restless legs syndrome (RLS). An increased prevalence of peripheral neuropathy has been demonstrated in Parkinson’s disease (PD). We aimed to investigate, in a cross-sectional manner, whether SFN is overrepresented in PD patients with concurrent RLS relative to PD patients without RLS, using in vivo corneal confocal microscopy (IVCCM) and quantitative sensory testing (QST) as part of small fiber assessment. Study participants comprised of age- and sex-matched PD patients with (*n* = 21) and without RLS (*n* = 21), and controls (*n* = 13). Diagnosis of RLS was consolidated with the sensory suggested immobilization test. Assessments included nerve conduction studies (NCS), Utah Early Neuropathy Scale (UENS), QST, and IVCCM, with automated determination of corneal nerve fiber length (CNFL) and branch density (CNBD) from wide-area mosaics of the subbasal nerve plexus. Plasma neurofilament light (p-NfL) was determined as a measure of axonal degeneration. No significant differences were found between groups when comparing CNFL (*p* = 0.81), CNBD (*p* = 0.92), NCS (*p* = 0.82), and QST (minimum *p* = 0.54). UENS scores, however, differed significantly (*p* = 0.001), with post-hoc pairwise testing revealing higher scores in both PD groups relative to controls (*p* = 0.018 and *p* = 0.001). Analysis of all PD patients (*n* = 42) revealed a correlation between the duration of l-dopa therapy and CNBD (*ρ* = −0.36, *p* = 0.022), and p-NfL correlated with UENS (*ρ* = 0.35, *p* = 0.026) and NCS (*ρ* = −0.51, *p* = 0.001). Small and large fiber neuropathy do not appear to be associated with RLS in PD. Whether peripheral small and/or large fiber pathology associates with central neurodegeneration in PD merits further longitudinal studies.

## Introduction

The prevalence of restless legs syndrome (RLS) in Parkinson’s disease (PD) has been reported as both higher^1,2^ and equal^3,4^ to that in the normal population. Diagnosis is made clinically, based on the characteristic history of a nocturnal urge to move the legs in order to relieve an often associated sensory discomfort^5^.

The pathogenesis of RLS is not fully understood, but studies have suggested different underlying pathophysiological mechanisms. These include disturbed cerebral iron metabolism, as assessed by imaging^6,7^, cerebrospinal fluid^8^ and pathological^9,10^ studies, and disruption of central dopaminergic pathways as evaluated by pathological studies^11^. Moreover, studies assessing peripheral small fiber function in RLS have reported conflicting results, with some favoring the presence of an RLS sub-phenotype associated with small fiber neuropathy (SFN)^12–14^.

An increased prevalence of small^15^ and large fiber^16,17^ neuropathy has been demonstrated in PD, suggested in part to reflect an underlying l-dopa-mediated disturbance of vitamin B12 metabolism^16,17^. However, considering reports of peripheral neuropathy in l-dopa naïve PD patients^18^, and the detection of deposits of alpha-synuclein (α-syn) in peripheral small nerve fibers^15^, neuropathy has also been proposed to reflect an intrinsic disease feature of PD.

In view of SFN as a possible contributing factor in the evolution of RLS, we conducted a cross-sectional study hypothesizing that PD with concurrent RLS is more highly associated with SFN compared to PD without RLS. As part of the assessment, we employed in vivo corneal confocal microscopy (IVCCM) as a novel technique to visualize small fiber morphology.

## Results

Fifty-nine participants were included, of which three in the PD with RLS (PD+RLS) group and one in the control group were excluded during the study period. Reasons for exclusion consisted of onset of stroke, bilateral cataract surgery, bilateral eye drop treatment, and detection of a pre-existing peripheral neuropathy when reviewing medical records. The final study population included 55 participants: PD+RLS (*n* = 21), PD without RLS (PD−RLS) (*n* = 21), and controls without PD (*n* = 13). The control group was smaller than initially planned, due to subjects declining to participate and/or fulfilling exclusion criteria.

### Baseline characteristics

The study groups were well matched in terms of age and sex (Table 1). All patients in the PD+RLS group fulfilled the International Restless Legs Syndrome Study Group (IRLSSG) criteria^5^ for the diagnosis of RLS as part of the inclusion criteria. Beyond this, a majority (81%) also exhibited a positive sensory suggested immobilization test (SIT)^19,20^. The severity of RLS symptoms in the PD+RLS group was severe, as reflected by a median score of 21 points on the IRLSSG rating scale (IRLS). The PD groups had comparable disease duration, levodopa equivalent daily dose (LEDD), and duration of l-dopa treatment. The median modified Hoehn and Yahr (mH&Y) stage was 2.0 in both PD groups, although a significantly higher mean rank was evident in the PD+RLS group (*p* = 0.039). In total, 45 participants underwent bilateral (17 PD+RLS, 17 PD−RLS, and 11 controls) and 10 unilateral (4 PD+RLS, 4 PD−RLS, and 2 controls) IVCCM. Baseline characteristics are outlined in Table 1.

**Table 1:** Demographic, clinical, and biochemical characteristics of the study population.

	PD+RLS (*n* = 21)	PD−RLS (*n* = 21)	CL (*n* = 13)	*p*
Age (years)	69.4 (5.9)	69.2 (6.0)	69.7 (6.6)	0.93^a^
Male/female	15/6	15/6	9/4	0.99^b^
Smoking (*n*, %yes)	3 (14.3)	2 (9.5)	2 (15.4)	0.89^c^
RLS heredity (*n*, %yes)	8 (38.1)	4 (19.0)	1 (7.7)	0.14^c^
PD heredity (*n*, %yes)	7 (33.3)	9 (42.9)	1 (7.7)	0.093^b^
B12 or multivitamins (*n*, %yes)	13 (61.9)	12 (57.1)	3 (23.1)	0.068^b^
B6 or multivitamins (*n*, %yes)	3 (14.3)	5 (23.8)	3 (23.1)	0.76^c^
Coffee consumption (cups/day)	2.5 (1.6)	2.2 (1.4)	3.7 (2.4)	0.12^a^
s-Ferritin (μg/L) [30–350]	159 (86.6)	159 (105)	186 (147)	0.94^a^
p-Homocysteine (μmol/L) [5–15]	15.9 (4.7)	15.0 (3.6)	15.5 (3.4)	1.0^a^
s-MMA (μmol/L) [<0.37]	0.18 (0.06)	0.17 (0.06)	0.17 (0.03)	0.90^a^
s-Folate (nmol/L) [>7]	22.8 (13.7)	19.6 (11.8)	18.2 (8.3)	0.67^a^
p-Pyridoxal-5ʹ-phosphate (nmol/L) [20–122]^d^	54.5 (32.7)	77.5 (68.9)	45.9 (26.5)	0.14^a^
p-NfL (pg/mL)	6.3 (3.8)	6.2 (4.2)	6.1 (4.1)	0.95^a^
*PD-specific variables*				
Motor duration (years)	7.9 (4.1)	7.5 (4.4)	–	0.66^e^
L-dopa duration (years)	5.1 (3.8)	4.6 (4.6)	–	0.42^e^
mH&Y (stage)	2.3 (0.4)	2.1 (0.5)	–	**0.039**^e^
LEDD (mg)	725 (360)	684 (308)	–	0.96^e^
No L-dopa treatment (*n*, %yes)	1 (4.8)	1 (4.8)	–	1.0^c^
*RLS-specific variables*				
RLS duration (years)	10.6 (11.3)	–	–	–
IRLS (p)	18.5 (7.9)	–	–	–
Positive SIT test^f^ (*n*, %yes)	17 (81.0)	–	–	–

Data are presented as mean (standard deviation) for numerical, and proportions (%) for categorical variables. Positive SIT was defined as a mean leg discomfort score >11. Heredity was defined as a positive family history of suspected PD or RLS.

*PD+RLS* Parkinson’s disease with restless legs syndrome, *PD−RLS* Parkinson’s disease without restless legs syndrome, *CL* controls, *B12/B6 or multivitamins* participants reporting intake of either multivitamins or vitamin B12/B6, *p* plasma, *s* serum, *MMA* methylmalonic acid, *NfL* neurofilament light, *mH&Y* modified Hoehn and Yahr, *LEDD* levodopa equivalent daily dose, *IRLS* International Restless Legs Syndrome Study Group Rating Scale, *SIT* suggested immobilization test.

In bold—indicates *p*-value ≤ 0.05.

^a^Kruskal–Wallis *H*-test.

^b^Chi-square test.

^c^Fisher’s exact test.

^d^In one blood sample, light protection was reported as insufficient.

^e^Mann–Whitney *U*-test.

^f^One participant reported discomfort from both arms and legs during assessment.

### Comparing peripheral nerve assessments between groups

**Fig. 1:**
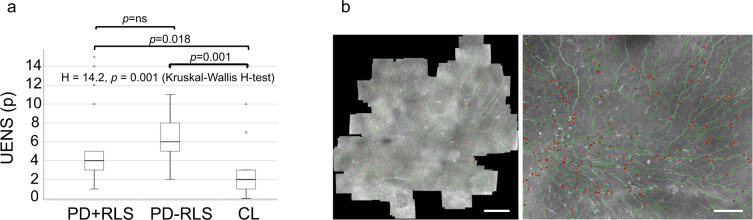
Group comparisons of UENS scores and representative mosaic image of the corneal subbasal nerve plexus. Comparison of UENS scores (**a**) between study groups. Boxplot showing center line (median), interquartile range (box length), whiskers (1.5 × interquartile range) and outliers. **b** Left: mosaic image from patient with PD and RLS showing traced nerve paths. Scale bar = 500 μm. Right: magnified region showing detailed nerve paths (green) and branching points (red). Scale bar = 100 μm.

A summary of the results from the battery of clinical, corneal, and electrophysiological assessments of small and large nerve fibers is reported in Table 2. A representative mosaic image of the corneal subbasal nerve plexus is shown in Fig. 1.

**Table 2:** Neurophysiological, corneal, and clinical assessments of peripheral neuropathy.

	PD+RLS (*n* = 21)	PD-RLS (*n* = 21)	CL (*n* = 13)	*p*^a^
*Clinical rating scale*				
UENS (p)	5.8 (4.1)	6.1 (2.4)	2.7 (2.8)	**0.001**
*Neurophysiological assessments*				
ENeG-Ix	−0.70 (0.95)	−0.64 (0.82)	−0.66 (0.64)	0.82
WT hand (°C)	2.6 (1.7)	2.4 (1.0)	2.4 (1.7)	0.84
CT hand (°C)	2.5 (1.9)	1.9 (1.1)	1.7 (0.47)	0.74
WT foot (°C)	10.9 (4.1)	10.8 (4.5)	9.7 (4.2)	0.71
CT foot (°C)	8.5 (10.0)	7.7 (6.9)	5.0 (2.8)	0.54
*In vivo corneal confocal microscopy*				
CNFL (mm/mm^2^)	17.5 (3.8)	16.9 (3.1)	17.6 (3.9)	0.81
CNBD (no/mm^2^)	105 (34.6)	106 (38.7)	111 (36.5)	0.92
Mature DCs (proportion, %)	16.0 (11.8)	18.0 (10.1)	21.5 (14.9)	0.56
Immature DCs (proportion, %)	69.5 (17.7)	70.1 (16.4)	72.5 (16.7)	0.89
Globular cells (proportion, %)	14.5 (16.2)	12.0 (15.6)	6.0 (4.2)	**0.050**
Mature DCs (density, cells/mm^2^)	6.1 (6.0)	9.0 (8.9)	10.7 (10.5)	0.58
Immature DCs (density, cells/mm^2^)	29.6 (24.8)	35.1 (29.9)	53.8 (51.4)	0.54
Globular cells (density, cells/mm^2^)	5.9 (10.4)	4.9 (9.0)	2.1 (1.4)	0.19

Data are presented as mean (standard deviation). No significant differences between PD patients with and without RLS were observed in the assessments of small fiber neuropathy. A higher UENS score was observed in PD groups relative to controls.

*PD+RLS* Parkinson’s disease with restless legs syndrome, *PD−RLS* Parkinson’s disease without restless legs syndrome, *CL* controls, *UENS* Utah Early Neuropathy Scale, *ENeG-Ix* electroneurography index, *WT* warmth threshold, *CT* cold threshold, *CNFL* corneal nerve fiber length, *CNBD* corneal nerve branch density, *DCs* dendritic cells.

In bold—indicates *p*-value ≤ 0.05.

^a^All analyses performed with Kruskal–Wallis *H*-test except CNFL, in which one-way ANOVA was used.

No group differences were detected with regard to corneal nerve fiber length (CNFL), (*p* = 0.81), or corneal nerve branch density (CNBD), (*p* = 0.92). A difference in scores on the Utah Early Neuropathy Scale (UENS) was observed between the three groups, in which post-hoc testing revealed lower UENS scores in controls relative to both PD groups after Bonferroni correction (PD+RLS: *p* = 0.018, PD-RLS: *p* = 0.001). However, no difference in UENS scores was demonstrated between PD+RLS and PD−RLS groups (*p* = 0.78) (Table 2 and Fig. 1). Assessment of small and large fiber function using quantitative sensory testing (QST) and nerve conduction studies (NCS) did not reveal any significant differences between the three study groups (Table 2).

Results from the quantification of cells in the subbasal corneal nerve plexus are shown in Table 2. No significant differences in the density of mature dendritic cells (DCs), immature DCs, and globular cells were seen between groups. When comparing the proportions of inflammatory cell types in the subbasal nerve plexus, a borderline-significant tendency (*p* = 0.050) was seen, suggesting a difference in cell composition between the groups, possibly favoring an increased proportion of globular cells in the PD+RLS group (Table 2).

Looking solely at PD patients that underwent bilateral IVCCM (*n* = 34), no significant differences in CNFL (*p* = 0.19) or CNBD (*p* = 0.10) were evident when comparing both eyes within-subjects, using the paired-samples *T*-test. Comparing PD patients with unilateral (*n* = 8) to PD patients with bilateral IVCCM (*n* = 34), no significant group differences were observed in mean CNFL (*p* = 0.44) or mean CNBD (*p* = 0.34).

### Associations between peripheral nerve assessments and PD burden

**Fig. 2:**
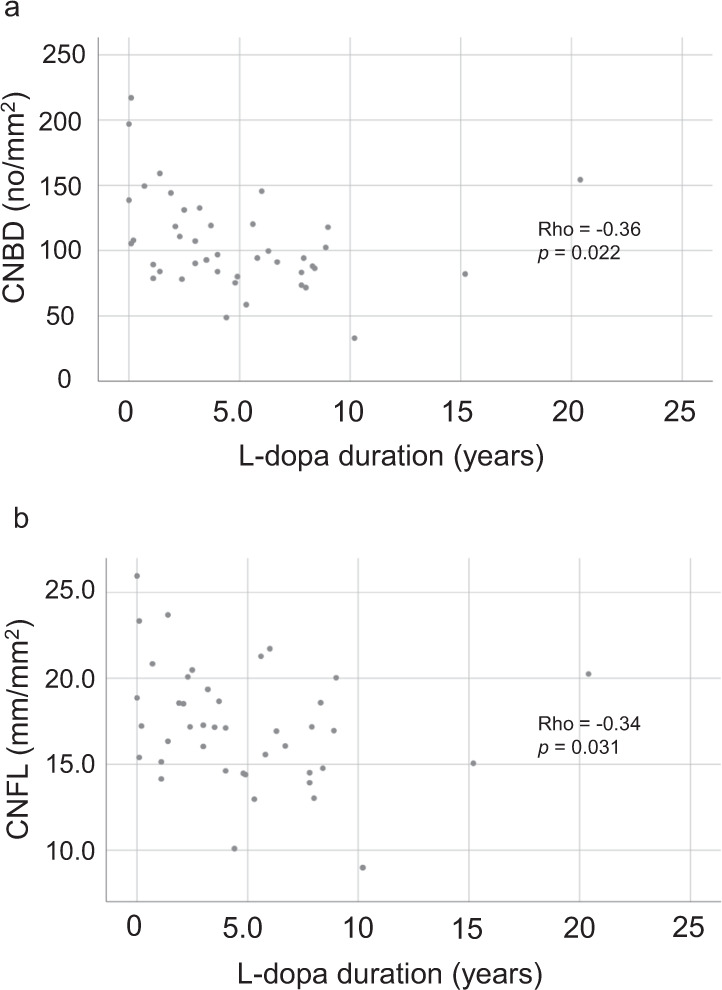
Associations between corneal parameters and l-dopa therapy. The duration of l-dopa therapy associates with CNBD (**a**) and CNFL (**b**). Correlation coefficients and *p*-values calculated with partial Spearman’s rank order correlation adjusting for age and sex (CNBD: *ρ*=−0.36, *p*=0.022), and age (CNFL: *ρ*=−0.34, *p*=0.031), respectively. *p*-Values not adjusted for multiple comparisons.

**Fig. 3:**
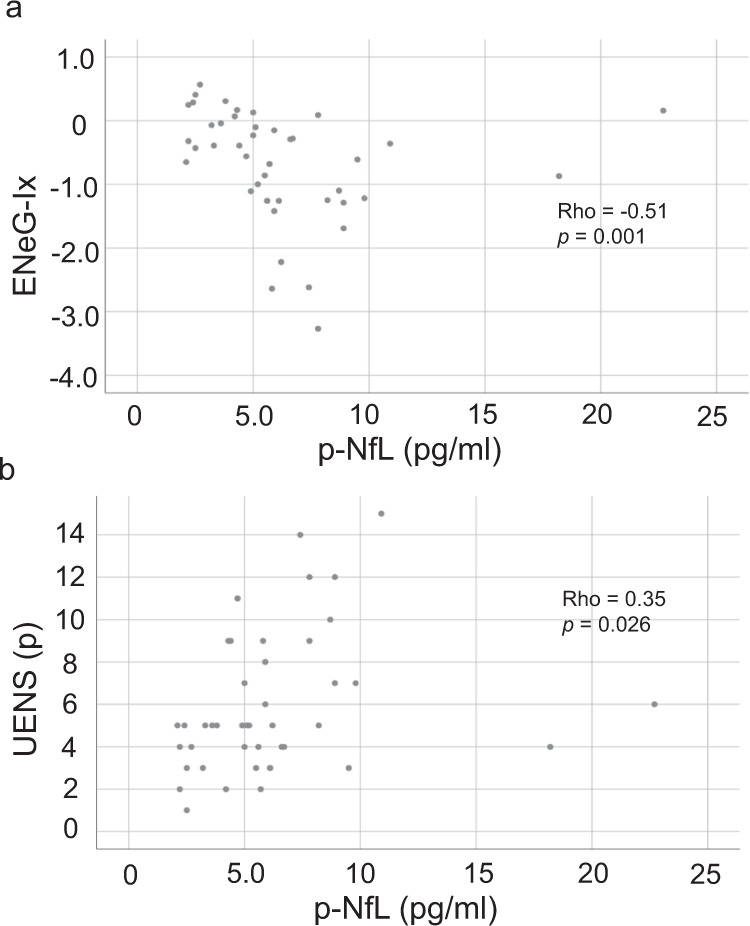
Associations between p-NfL and functional peripheral nerve parameters. Associations between measures of large fiber function, as assessed by nerve conduction studies (**a**) and UENS (**b**), and p-NfL. Correlation coefficients and *p*-values calculated with partial Spearman’s rank order correlation adjusting for age and sex (ENeG-Ix: *ρ*=−0.51, *p*=0.001; UENS: *ρ*=0.35, *p*=0.026). *p*-Values not adjusted for multiple comparisons.

To assess potential associations between peripheral nerve fiber pathology and indirect measures of overall PD burden, a subgroup analysis was performed in all PD patients (*n* = 42). Using the age-adjusted partial Spearman’s rank correlation test, an association was found between corneal parameters (CNFL and CNBD) and the duration of l-dopa therapy (*ρ* = −0.34, *p* = 0.031 and *ρ* = −0.36, *p* = 0.022, respectively); however, only the association with CNBD was significant when controlling for both age and sex (Fig. 2). A correlation, after adjusting for age and sex, was also observed between the electroneurography index (ENeG-Ix) and plasma neurofilament light (p-NfL) (*ρ* = −0.51, *p* = 0.001, Fig. 3a). Moreover, p-NfL exhibited an age- and sex-adjusted correlation with UENS scores (*ρ* = 0.35, *p* = 0.026), (Fig. 3b). Finally, the mH&Y stage correlated significantly with the warmth (*ρ* = 0.35, *p* = 0.028) and cold (*ρ* = 0.37, *p* = 0.019) thresholds of the hand, adjusting for age and sex. All tested correlations are summarized in Supplementary Table 1.

### Markers of methionine cycle metabolism

No significant group differences were observed when comparing levels of p-pyridoxal-5-phosphate (vitamin B6), s-methylmalonic acid (MMA), s-folate, and p-homocysteine (Table 1). The median levels of these parameters were all within the normal range in patients with PD. Notably, a considerable proportion (60%) of patients with PD reported intake of vitamin B12 or multivitamin supplements.

A subgroup analysis was performed in the entire PD group (*n* = 42), comparing patients with (*n* = 25) and without (*n* = 17) any B-vitamin supplementation. No significant differences with regard to peripheral nerve parameters were demonstrated between the two groups (Table 3). Notable significant differences were evident with regard to the duration of l-dopa therapy (*p* = 0.002) and disease duration (*p* = 0.001), suggesting a more advanced disease in patients receiving B-vitamin supplementation. Demographic data together with clinical, corneal, and electrophysiological data for these two groups are shown in Table 3.

**Table 3:** Characteristics of subgroup analysis comparing PD patients with and without B-vitamin supplementation.

	PD+B-vit. (*n* = 25)	PD−B-vit. (*n* = 17)	*p*
Age (years)	69.1 (5.4)	69.6 (6.6)	0.53^a^
Male/female	19/6	11/6	0.50^b^
p-Homocysteine (μmol/L) [5–15]	14.6 (4.0)	16.7 (4.3)	0.078^a^
p-NfL (pg/mL)	7.0 (4.6)	5.1 (2.6)	0.11^a^
*PD-specific variables*			
Motor duration (years)	9.2 (4.3)	5.6 (3.0)	**0.001**^a^
l-dopa duration (years)	6.2 (4.3)	2.8 (3.0)	**0.002**^a^
mH&Y (stage)	2.3 (0.48)	2.1 (0.42)	0.22^a^
LEDD (mg)	749 (285)	640 (390)	0.12^a^
No l-dopa treatment (*n*, %yes)	0 (0)	2 (11.8)	0.16^b^
RLS study diagnosis (*n*, %yes)	13 (52.0)	8 (47.1)	0.75^c^
*Peripheral nerve parameters*			
UENS (p)	6.1 (3.3)	5.8 (3.4)	0.70^a^
ENeG-Ix	−0.85 (0.95)	−0.41 (0.72)	0.10^a^
WT hand (°C)	2.7 (1.6)	2.3 (1.1)	0.73^a^
CT hand (°C)	2.3 (1.8)	2.0 (1.0)	0.91^a^
WT foot (°C)	11.7 (3.9)	9.6 (4.5)	0.21^a^
CT foot (°C)	8.9 (9.4)	7.0 (7.2)	0.56^a^
CNFL (mm/mm^2^)	17.0 (3.1)	17.6 (4.0)	0.57^d^
CNBD (no/mm^2^)	103 (28.0)	110 (46.5)	0.52^d^

Data are presented as mean (standard deviation) for numerical, and proportions (%) for categorical variables. A significantly longer disease duration and duration of l-dopa exposure was observed in the group receiving vitamin B supplements. No significant differences were observed with regard to peripheral nerve parameters.

*PD+B-vit* Parkinson’s disease with vitamin B6 and/or B12 and/or multivitamin supplementation, *PD−B-vit.* Parkinson’s disease without vitamin B supplementation, *NfL* neurofilament light, *mH&Y* modified Hoehn and Yahr, *LEDD* levodopa equivalent daily dose, *UENS* Utah Early Neuropathy Scale, *ENeG-Ix* electroneurography index, *WT* warmth threshold, *CT* cold threshold, *CNFL* corneal nerve fiber length, *CNBD* corneal nerve branch density.

In bold—indicates *p*-value ≤ 0.05.

^a^Mann–Whitney *U*-test.

^b^Fisher’s exact test.

^c^Chi-square test.

^d^Independent *T*-test.

## Discussion

The main finding of this study is that SFN, as assessed by IVCCM, QST, and UENS, appears not to be associated with RLS in PD. Furthermore, we could neither demonstrate any association between RLS in PD and large fiber neuropathy, as assessed by NCS (Table 2).

A previous study also addressed a possible association between peripheral neuropathy and PD with RLS in a cross-sectional manner, similarly with negative findings^21^. However, the assessments in that study consisted of NCS and UENS, without further evaluation of small fiber function or morphology^21^. Since SFN has been proposed as an intrinsic feature of PD^15^, we believe the present study adds further knowledge to the possible clinical correlates of SFN in PD. In other words, RLS does not seem to represent a phenotypic expression of SFN in PD.

An important part of the present study was to ensure a reliable diagnosis of RLS. RLS mimics, such as leg cramps, akathisia, inner restlessness, and wearing-off phenomenon, are important to differentiate when diagnosing RLS in PD^22^. In the present study, all PD+RLS patients fulfilled the IRLSSG criteria^5^, and common pharmacological and metabolic triggers of RLS were part of the study exclusion criteria. Furthermore, the sensory SIT was employed in the PD+RLS group, reaching a positive outcome in 81% of the patients (Table 1). The sensory SIT has been shown to have a 91% sensitivity and 72% specificity for RLS in the context of PD, when performed during symptomatic RLS periods^20^. Considering the high probability that not every PD+RLS patient was in an active symptom period at the time of testing, we believe the 81% test positivity is supportive of a true RLS diagnosis in the PD+RLS group. We also believe the comprehensive diagnostic evaluation performed in the PD+RLS group consolidates the reliability of the main study result, that RLS in PD appears not to be associated with small or large fiber neuropathy.

In the present study, we could not detect any significant differences in measures of peripheral neuropathy in patients with PD, as assessed by IVCCM, QST, and NCS, relative to controls. However, a significantly higher UENS score was seen in both PD groups relative to controls (Table 2). These results are in contrast to previous studies, where an increased prevalence of large fiber neuropathy in PD has been reported, when evaluated with both NCS and clinical rating scales^16,23,24^. In those studies, neuropathy was suggested to be associated with alterations in the vitamin B12-dependent methionine cycle, mediated by chronic exposure to l-dopa and thus associated with elevated levels of p-homocysteine and s-MMA^16,23,24^. In the present study, 60% of the PD patients were taking vitamin B12 or multivitamin supplements, and the median levels of p-homocysteine and s-MMA were within normal range. In order to address possible confounding effects of B-vitamin supplementation, a subgroup analysis comprising all PD patients (*n* = 42) was performed. As outlined in Table 3, no significant differences in corneal, electrophysiological or clinical assessments of peripheral nerves were found between PD patients with and without B-vitamin supplementation. However, a significantly longer disease duration (*p* = 0.001) and duration of l-dopa exposure (*p* = 0.002) was evident in the B-vitamin supplemented group. Thus, we still cannot exclude the presence of a protective, and confounding, effect of B-vitamin supplementation that contributed to the absence of a significantly higher prevalence of peripheral neuropathy in the more advanced disease group. This finding may be of interest, and motivates further investigation of possible protective effects of vitamin B12 supplementation with regard to the development of peripheral neuropathy in PD.

SFN has been demonstrated in skin biopsies from l-dopa naïve patients^18^. Furthermore, cutaneous SFN in PD has, in some studies, been reported as asymmetrical, lateralizing with the side more affected by parkinsonism^18,25^. Considering these studies, together with the reported findings of α-syn deposits in autonomic and somatosensory small nerve fibers, the concept of peripheral neurodegeneration intrinsic to PD has been suggested^15^. Surprisingly in the present study, in line with findings reported for large fiber neuropathy, no significant differences in SFN, as assessed by IVCCM and QST, were found in the PD groups relative to controls. Limitations of this study, however (discussed below), must be considered.

The human cornea is heavily innervated by small C- and Aδ nerve fibers, originating from the trigeminal nerve^26^. In diabetes mellitus, the visualization of small nerve fiber pathology in the corneal subbasal nerve plexus has been proposed as a surrogate marker of general diabetic peripheral neuropathy^27^. Here, we chose to present large mosaic depictions of the subbasal nerve plexus. Recent studies have proposed imaging and analysis of a wide area of the subbasal nerve plexus to be advantageous, by reducing inherent biases associated with subjectively imaging, and selecting, typically only a few single microscope frames for nerve analysis (each frame representing 0.2% of the total area of the subbasal nerve plexus). In a study examining patients with multiple sclerosis, mosaics with a mean size of 1.29 mm^2^ (corresponding to the area of 7.7 individual microscope frames) were produced^28^. In a previous study with healthy and type 2 diabetes participants, we reported a mean mosaic size of 6.0 mm^2^ (37 frames)^29^, whereas in the present study the mean mosaic size was 7.7 mm^2^ (48 frames). Moreover, the present IVCCM methods were fully automated, avoiding observer-dependent biases in image selection and analysis.

Only a few previous studies have assessed corneal nerve parameters in PD, with conflicting results. A study investigating 26 patients with PD, with varying disease duration, detected significantly increased CNBD and CNFL relative to controls, correlating negatively with motor scores and autonomic symptoms^30^. In that study, 4–6 single confocal microscope image frames per eye (non-mosaic) were manually selected. By contrast, in a study consisting of 26 early PD patients with minimal l-dopa exposure, significantly decreased CNBD and CNFL were reported relative to controls, with the authors proposing the corneal alterations may reflect a preclinical neuropathy in PD^31^. In that study, 4–8 single images frames per eye were manually selected for analysis.

The present study did not confirm these prior discriminative findings in corneal parameters with respect to controls, but instead detected similar CNBD and CNFL in all three groups (Table 2). Even when comparing controls to the PD group as a whole (*n* = 42), no differences were detected (CNFL: *p* = 0.84; CNBD: *p* = 0.71). Importantly, the present study was not primarily designed to assess the power of CNBD and CNFL to discriminate between PD and controls. Nevertheless, in the context of previous studies in PD, we believe a strength of the present study is the use of large mosaic depictions of the subbasal nerve plexus. Prior studies examining PD patients with IVCCM quantified 10–12% of the subbasal plexus area quantified in this study, and used manual image selection and semi-manual nerve quantification methods. Therefore, we believe the present study, using robust methodology, suggests that CNBD and CNFL are not suitable as discriminative diagnostic assessments in moderate PD.

In diabetes mellitus, an increased proportion of mature DCs in the subbasal nerve plexus has been reported and was suggested to reflect a corneal immune-activation associated with diabetic disease^32^. The present study provided no evidence for immune activation in the corneal subbasal nerve plexus in patients with PD. The least prevalent cell type detected in all groups were the globular cells. Although the median globular cell density was similar among groups, the Kruskal–Wallis *H*-test indicated a borderline-significant (*p* = 0.050) proportional difference between the three groups, possibly suggestive of an increased relative proportion of globular cells in the PD+RLS group (Table 2). The biological implication of this finding is uncertain, and as of now, the role of this cell type is not known.

Peripheral neuropathy has been suggested as an independent marker of a more severe PD phenotype, associated with an increased burden of both motor and non-motor symptoms^33^. In the subgroup analysis (*n* = 42), we examined if measures of small and/or large fiber pathology were associated with indirect markers of general disease progression (Figs. 2 and 3). We believe the demonstrated associations between corneal parameters and the duration of l-dopa therapy may merit future studies evaluating the potential of monitoring small fiber morphology, as assessed by IVCCM, as a marker of ongoing central neurodegeneration. However, in such studies it will be important to account for confounding effects of L-dopa-mediated alterations of the methionine cycle. Thus, inclusion of careful analyses of vitamin B6, B12, s-MMA, and p-homocysteine are required; in the present study, 60% of patients with PD were taking supplements and median p-homocysteine levels were normal (Table 1).

Plasma NfL is a marker of axonal degeneration^34^, and has been suggested to be associated with both PD progression^35^ and disease activity in hereditary peripheral neuropathy^36,37^. Indeed, significant associations were demonstrated in the present study between p-NfL and ENeG-Ix and UENS, even after adjusting for age and sex (Fig. 3). Considering p-NfL also correlated significantly with mH&Y, after adjustment for age and sex (*ρ* = 0.39, *p* = 0.013), we believe p-NfL might reflect both central and peripheral ongoing axonal neurodegeneration in PD.

The small study group constitutes the main limitation of this study. Importantly, the control group was smaller than the two PD groups, and as a result, comparisons relative to controls might have both under- and overestimated group differences. However, the main aim of this study was to assess whether SFN is overrepresented in PD+RLS relative to PD−RLS, and thus we believe our main finding was not affected by the smaller control group. Considering reports of asymmetrical presentations of SFN in PD^18,25^, the unilateral QST, in contrast to the bilateral UENS, might have underestimated SFN in PD. Since the tested side was randomly chosen, this should not have affected comparisons between PD groups but rather comparisons relative to controls. Similarly, the randomly chosen side for motor and sensory NCS might also have contributed to the absence of large fiber neuropathy relative to controls in the entire PD group. The reliability of RLS diagnosis is important when interpreting the main study results. As discussed, four patients exhibited a negative sensory SIT and thus a repeated test, during a symptomatic period, could have been done to further consolidate the diagnosis in these patients. The subgroup analysis, encompassing all PD patients (*n* = 42), did not constitute the main aim of this study and was thus considered explorative in nature. Therefore, Bonferroni adjustments for multiple comparisons in the correlation analyses were not performed and as such, type 1 errors must be taken into consideration when interpreting these data.

RLS in PD does not appear to be associated with small or large fiber neuropathy as assessed by IVCCM, QST, UENS, and NCS. The potential of objective functional and structural assessments of peripheral small and large fibers, as a surrogate marker of PD progression, warrants further evaluation in longitudinal studies accounting for both the reported asymmetrical presentations of peripheral neuropathy in PD, and the possible confounding role of disturbed methionine cycle metabolism attributed to l-dopa exposure.

## Methods

### Participants

All participants gave written informed consent and the study was approved by the regional ethical board of Stockholm, Sweden (ref. nr 2018/264-31/2 (2019-03158)). Patient-related investigations were undertaken in accordance with the Helsinki Declaration.

Participants were recruited between the spring of 2018 and autumn of 2019. Patients with PD followed at the outpatient clinic at Center for Neurology and Karolinska University Hospital, Stockholm, were invited to participate if reporting symptoms indicative of RLS. We also used a written advertisement, posted at the local patients’ organization website, inviting patients with PD and RLS symptoms from the Stockholm region to participate. Patients meeting criteria were included and constituted the PD+RLS group. Controls and PD patients not meeting RLS criteria, and matched for age, sex, and disease duration, were also invited to participate during visits to the outpatient clinic. All participants were aged 50–80 years and had at least one eye free from previous corneal trauma, surgery, or ongoing eye drop treatment. Accompanying persons or spouses constituted the control group.

Inclusion criteria for patients consisted of a diagnosis of clinically probable PD according to the Movement Disorders Society criteria^38^ and RLS according to the IRLSSG criteria^5^ where applicable. Exclusion criteria included a known diagnosis of diabetes mellitus, rheumatoid arthritis, polyneuropathy, iron deficiency anemia, or renal failure (p-creatinine >150 µmol/L); heavy alcohol consumption (≥168 (men) or ≥108 (women) g alcohol/week)^39^; ongoing medication with selective serotonin reuptake inhibitors, serotonin–norepinephrine reuptake inhibitors, tricyclic antidepressants, or neuroleptic drugs at the time of RLS onset.

### Clinical assessments

Clinical information including smoking habits, alcohol consumption, heredity, and current medication was obtained through oral history and review of medical records. LEDD was calculated as previously described^40^. Disease duration was defined as time since motor symptom onset. Clinical examination included mH&Y^41,42^ staging and the UENS^43^, a clinical rating scale sensitive for the detection of SFN. The severity of RLS symptoms was evaluated with the IRLS^44^.

### Biochemistry

Fasting venous blood samples were collected and analyzed at Karolinska University Laboratory according to clinical routine. The tests included p-homocysteine, s-MMA, s-folate, s-cobalamin, s-ferritin, p-glucose, and b-HbA1c. Analysis of p-pyridoxal-5-phosphate (vitamin B6) was performed at Sahlgrenska University Hospital, Gothenburg. Plasma NfL concentration was measured using an in-house single molecule array (Simoa) assay, as described previously in detail^45^, at Sahlgrenska University Hospital, Mölndal. All p-NfL analyses were performed in one run, using the same batch of reagents, by board-certified laboratory technicians blinded to the clinical information.

### Sensory suggested immobilization test

The PD+RLS group was further assessed with the sensory SIT^19,20^. Patients were observed in the evening, between 8 PM and 9 PM, lying down in a 45° recumbent position and instructed to move as little as possible with legs extended. Patients were asked every 10 min to indicate their perceived severity of leg discomfort, using a visual analog scale of 0–100, generating seven individual values for each participant. A mean leg discomfort score >11 was used as supportive of RLS diagnosis. This cutoff value has previously been evaluated and proposed as appropriate in the context of RLS diagnosis in PD^20^.

### Neurophysiology

Electrodiagnostic testing and QST took place at the Department of Neurophysiology, Karolinska University Hospital. Motor NCS were carried out unilaterally in the median, peroneal, and tibial nerves, and sensory NCS unilaterally in the median and sural nerves with surface electrodes, using Viking EDX (Cephalon A/S; Denmark). The tested side was chosen randomly and care was taken to make all recordings at a skin temperature of >32 °C. Twelve parameters were chosen to calculate an index (ENeG-Ix) as previously described^46^. In short, six parameters represent conduction velocities (3 motor + 3 sensory, 3 upper + 3 lower extremity) and six represent amplitudes (3 compound muscle action potentials + 3 sensory nerve action potentials, 3 upper + 3 lower extremity). To achieve a more Gaussian distribution, the natural logarithms of the amplitudes were used. The ENeG-Ix is then calculated as the mean deviation (in SD) from normal controls standardized for age and height. The ENeG-Ix thus reflects peripheral large fiber function, correlating negatively with the degree of neuropathy. An index value that differs >0.72 SD from normal is considered abnormal. For details in ENeG-Ix calculation, see Solders et al.^46^.

QST was performed by the method of levels unilaterally over the thenar muscles in the hand and on the lateral part of the foot using Medusa TSA II (Cephalon A/S; Denmark). The probe operating by the Peltier principle has a rectangular surface of 2.5 × 5.0 cm. The baseline temperature of the probe was set to 32 °C. Five cold and five warm stimuli were delivered with a rise or fall in temperature of 1 °C/s. The participants were instructed to press a handheld button as soon as she/he experienced a sensation of cold or warmth, thereby also returning the probe to 32 °C. The perceptions thresholds were determined as the difference between 32 °C and the mean perception level of the five stimuli for cold (CT) and warmth (WT), respectively.

### In vivo corneal confocal microscopy

All participants underwent IVCCM bilaterally, or unilaterally if one eye met exclusion criteria. The central corneal subbasal nerve plexus was imaged as previously described^47^. Briefly, a topically anesthetized eye was examined with the Heidelberg Retinal Tomograph 3 laser-scanning confocal microscope with the Rostock Corneal Module (Heidelberg Engineering, Germany). A single examiner performed all eye scanning, recording images of the subbasal nerve plexus across a wide area of the plexus using the built-in fixation light to access paracentral regions and continually adjusting the focus to the plexus depth. Mosaics were generated with an automated computer algorithm to select nerve plexus images from the recorded data using tissue classification^48^ and to stitch together adjacent images. Depth variations of subbasal nerve fiber paths were mapped onto a single two-dimensional mosaic image^47^. A separate automated algorithm was used for detection and tracing of nerve paths and branching points, from which the mean values of CNFL (total nerve fiber length in a mosaic divided by the mosaic area, expressed in mm/mm^2^) and CNBD (total number of nerve branching points divided by the mosaic area, expressed as the number of branching points per mm^2^) were calculated^49,50^. Averaged values between both eyes were used where applicable.

In addition, two independent trained observers performed a morphological characterization and manual quantification of cells present in the subbasal nerve plexus. All cells present in the mosaics were counted and classified by both observers, purely by visual morphology as mature DCs, immature DCs, and globular cells, as previously described^32^. The quantitative results were averaged between the two observers and across both eyes per participant where applicable, generating proportional and density data for each cell type in each study participant. The observers were masked to the identity of each mosaic image.

### Statistical analysis

Categorical variables are presented as proportions. The distribution of continuous variables was tested for normality by using the Shapiro-Wilk test and assessment of skewness. Numerical variables are presented as mean (standard deviation). For comparison between groups, Mann–Whitney *U*-test and Kruskal–Wallis *H*-test were used for numerical variables not meeting assumptions for parametric testing. Independent *T*-test and one-way analysis of variance was used for comparison of normally distributed variables between groups, when the assumption of homoscedasticity was met. The paired-samples *T*-test was performed in dependent observations, after analyzing the differences between the dependent variables. In the absence of outliers and when normal distribution of the differences was evident, as assessed by skewness and the Shapiro-Wilk test, the paired *T*-test was performed. Categorical variables were compared using chi-square test, and Fisher’s exact test when the assumption of minimum expected values was not met. Correlation analyses for non-normally distributed variables were done using partial Spearman’s rank order correlation. A two-tailed *p*-value of < 0.05 was considered significant. All statistical analyses were performed using IBM SPSS Statistics for Windows, version 25.0 (IBM Corp., Armonk, N.Y., USA).

### Reporting summary

Further information on research design is available in the Nature Research Reporting Summary linked to this article.

## Supplementary information

Supplementary Table 1

Reporting Summary Checklist

## Data Availability

Anonymized data not published within this article will be shared upon request from any qualified investigator.
