# Supplementary material for: Parkinson’s disease with restless legs syndrome—an in vivo corneal confocal microscopy study
Source: NPJ Parkinsons Dis. 2021 Jan 5;7:4. doi: 10.1038/s41531-020-00148-5 (PMC7785738; doi:10.1038/s41531-020-00148-5)
Supplement: Supplementary file 1 — Supplementary Table 1 [file 41531_2020_148_MOESM1_ESM.pdf]

Supplementary TABLE 1.
Associations between peripheral nerve fiber parameters and indirect measures of PD burden in the whole PD group (n=42).

	CNFL (mm/mm^2)	CNBD (no/mm^2)	UENS (p)	ENeG-Ix	WT/CT hand (◦C)	WT/CT foot (◦C)
PD duration (years)	ns	ns	ns	ns	ns	ns
L-dopa duration (years)	-0.34*[2]	-0.36**[1]	ns	ns	ns	ns
LEDD (mg)	ns	ns	ns	ns	ns	ns
mH&Y	ns	ns	ns	ns	0.35*[1] / 0.37**[1]	ns
p-NfL (pg/ml)	ns	ns	0.35*[1]	-0.51***[1]	ns	ns

Abbreviations: CNFL – corneal nerve fiber length; CNBD – corneal nerve branch density; UENS – Utah Early Neuropathy Scale; ENeG-Ix – electroneurography index; WT – warmth threshold; CT – cold threshold; LEDD – levodopa equivalent daily dose; mH&Y – modified Hoehn and Yahr; NfL – neurofilament light; ns – *p* not significant
*p<0.05, **p<0.025, ***p=0.001
[1]Adjusted for age and sex, [2]Adjusted only for age

Table legend 1.

Correlation coefficients are shown for significant associations, as calculated with partial Spearman's rank order correlation. P-values not adjusted for multiple comparisons.
