# Supplementary material for: Parkinson’s disease with restless legs syndrome—an in vivo corneal confocal microscopy study
Source: NPJ Parkinsons Dis. 2021 Jan 5;7:4. doi: 10.1038/s41531-020-00148-5 (PMC7785738; doi:10.1038/s41531-020-00148-5)
Supplement: Supplementary file 2 — Reporting Summary Checklist [file 41531_2020_148_MOESM2_ESM.pdf]

nature research

Corresponding author(s): Mattias Andréasson

Last updated by author(s): Nov 1, 2020

# Reporting Summary

Nature Research wishes to improve the reproducibility of the work that we publish. This form provides structure for consistency and transparency in reporting. For further information on Nature Research policies, see our Editorial Policies and the Editorial Policy Checklist.

## Statistics

For all statistical analyses, confirm that the following items are present in the figure legend, table legend, main text, or Methods section.

| n/a | Confirmed | |
|---|---|---|
| ☐ | ☒ | The exact sample size (*n*) for each experimental group/condition, given as a discrete number and unit of measurement |
| ☐ | ☒ | A statement on whether measurements were taken from distinct samples or whether the same sample was measured repeatedly |
| ☐ | ☒ | The statistical test(s) used AND whether they are one- or two-sided<br>*Only common tests should be described solely by name; describe more complex techniques in the Methods section.* |
| ☐ | ☒ | A description of all covariates tested |
| ☐ | ☒ | A description of any assumptions or corrections, such as tests of normality and adjustment for multiple comparisons |
| ☐ | ☒ | A full description of the statistical parameters including central tendency (e.g. means) or other basic estimates (e.g. regression coefficient) AND variation (e.g. standard deviation) or associated estimates of uncertainty (e.g. confidence intervals) |
| ☐ | ☒ | For null hypothesis testing, the test statistic (e.g. *F*, *t*, *r*) with confidence intervals, effect sizes, degrees of freedom and *P* value noted<br>*Give P values as exact values whenever suitable.* |
| ☒ | ☐ | For Bayesian analysis, information on the choice of priors and Markov chain Monte Carlo settings |
| ☒ | ☐ | For hierarchical and complex designs, identification of the appropriate level for tests and full reporting of outcomes |
| ☒ | ☐ | Estimates of effect sizes (e.g. Cohen's *d*, Pearson's *r*), indicating how they were calculated |

*Our web collection on statistics for biologists contains articles on many of the points above.*

## Software and code

Policy information about availability of computer code

| | |
|---|---|
| Data collection | No specific code was used to collect data. |
| Data analysis | All statistical analyses were performed using IBM SPSS Statistics for Windows, version 25.0 (IBM Corp., Armonk, N.Y., USA). |

For manuscripts utilizing custom algorithms or software that are central to the research but not yet described in published literature, software must be made available to editors and reviewers. We strongly encourage code deposition in a community repository (e.g. GitHub). See the Nature Research guidelines for submitting code & software for further information.

## Data

Policy information about availability of data

All manuscripts must include a data availability statement. This statement should provide the following information, where applicable:

- Accession codes, unique identifiers, or web links for publicly available datasets
- A list of figures that have associated raw data
- A description of any restrictions on data availability

Anonymized data not published within this article will be shared upon request from any qualified investigator.

# Field-specific reporting

Please select the one below that is the best fit for your research. If you are not sure, read the appropriate sections before making your selection.

☒ Life sciences ☐ Behavioural & social sciences ☐ Ecological, evolutionary & environmental sciences

For a reference copy of the document with all sections, see nature.com/documents/nr-reporting-summary-flat.pdf

# Life sciences study design

All studies must disclose on these points even when the disclosure is negative.

| | |
|---|---|
| Sample size | With regard to the clinical rating scale (UENS) a calculation was done before the study started. We hypothesized a mean 7 points (SD 4p) on the UENS scale for the PD+RLS group, and 3 points (SD 4p) in the PD-RLS group. In order to demonstrate a significant (p<0.05) difference between these two groups with >80 % power we would need 16 participants in each group. |
| Data exclusions | 59 participants were included in the study, of which three in the PD with RLS (PD+RLS) group and one in the control group were excluded during the study period. Reasons for exclusion consisted of onset of stroke, bilateral cataract surgery, bilateral eye drop treatment and detection of a pre-existing peripheral neuropathy when reviewing medical records. |
| Replication | Not applicable for the present study |
| Randomization | The study aimed to compare patients with PD + RLS to PD without RLS. Thus, this was not a clinical trial why no randomization was done. The three groups (controls included) were matched with regard to age, gender and disease duration. |
| Blinding | When analyzing the corneal morphological data, the observers were masked to the identity of each mosaic image. When |

# Reporting for specific materials, systems and methods

We require information from authors about some types of materials, experimental systems and methods used in many studies. Here, indicate whether each material, system or method listed is relevant to your study. If you are not sure if a list item applies to your research, read the appropriate section before selecting a response.

## Materials & experimental systems

| n/a | Involved in the study | |
|---|---|---|
| ☒ | ☐ | Antibodies |
| ☒ | ☐ | Eukaryotic cell lines |
| ☒ | ☐ | Palaeontology and archaeology |
| ☒ | ☐ | Animals and other organisms |
| ☐ | ☒ | Human research participants |
| ☒ | ☐ | Clinical data |
| ☒ | ☐ | Dual use research of concern |

## Methods

| n/a | Involved in the study | |
|---|---|---|
| ☒ | ☐ | ChIP-seq |
| ☒ | ☐ | Flow cytometry |
| ☒ | ☐ | MRI-based neuroimaging |

## Human research participants

Policy information about studies involving human research participants

| | |
|---|---|
| Population characteristics | Patients with PD were invited to participate if reporting symptoms indicative of RLS. Patients meeting criteria were included and constituted the PD+RLS group. Controls and PD patients not meeting RLS criteria, and matched for age, sex and disease duration, were also invited to participate. All participants were aged 50-80 years and had at least one eye free from previous corneal trauma, surgery or ongoing eye drop treatment. Accompanying persons or spouses constituted the control group. Inclusion criteria for patients consisted of a diagnosis of clinically probable PD according to the Movement Disorders Society criteria and RLS according to the IRLSSG criteria where applicable. Exclusion criteria included a known diagnosis of diabetes mellitus, rheumatoid arthritis, polyneuropathy, iron deficiency anemia or renal failure (p-creatinine >150 µmol/l); heavy alcohol consumption (≥168 (men) or ≥108 (women) g alcohol/week)39; ongoing medication with selective serotonin reuptake inhibitors, serotonin-norepinephrine reuptake inhibitors, tricyclic antidepressants or neuroleptic drugs at the time of RLS onset. |
| Recruitment | Participants were recruited between the spring of 2018 and autumn of 2019. Patients with PD followed at the outpatient clinic at Center for Neurology and Karolinska University Hospital, Stockholm, were invited to participate if reporting symptoms indicative of RLS. We also used a written advertisement, posted at the local patients' organization website, inviting patients with PD and RLS symptoms from the Stockholm region to participate.<br>Controls and PD patients not meeting RLS criteria, and matched for age, sex and disease duration, were also invited to participate during visits to the outpatient clinic. All participants were aged 50-80 years and had at least one eye free from |


April 2020

| | |
|---|---|
| | previous corneal trauma, surgery or ongoing eye drop treatment. Accompanying persons or spouses constituted the control group. |
| Ethics oversight | All participants gave written informed consent and the study was approved by the regional ethical board of Stockholm, Sweden (ref. nr 2018/264-31/2 (2019-03158)). |

Note that full information on the approval of the study protocol must also be provided in the manuscript.
